# First person – Monika Baxa

**DOI:** 10.1242/dmm.043372

**Published:** 2019-12-12

## Abstract

First Person is a series of interviews with the first authors of a selection of papers published in Disease Models & Mechanisms, helping early-career researchers promote themselves alongside their papers. Monika Baxa is first author on ‘Longitudinal study revealing motor, cognitive and behavioral decline in a transgenic minipig model of Huntington's disease’, published in DMM. Monika is a PhD student in the lab of Zdenka Ellederova at the Czech Academy of Sciences, Czech Republic, investigating pig models of neurodegenerative diseases.


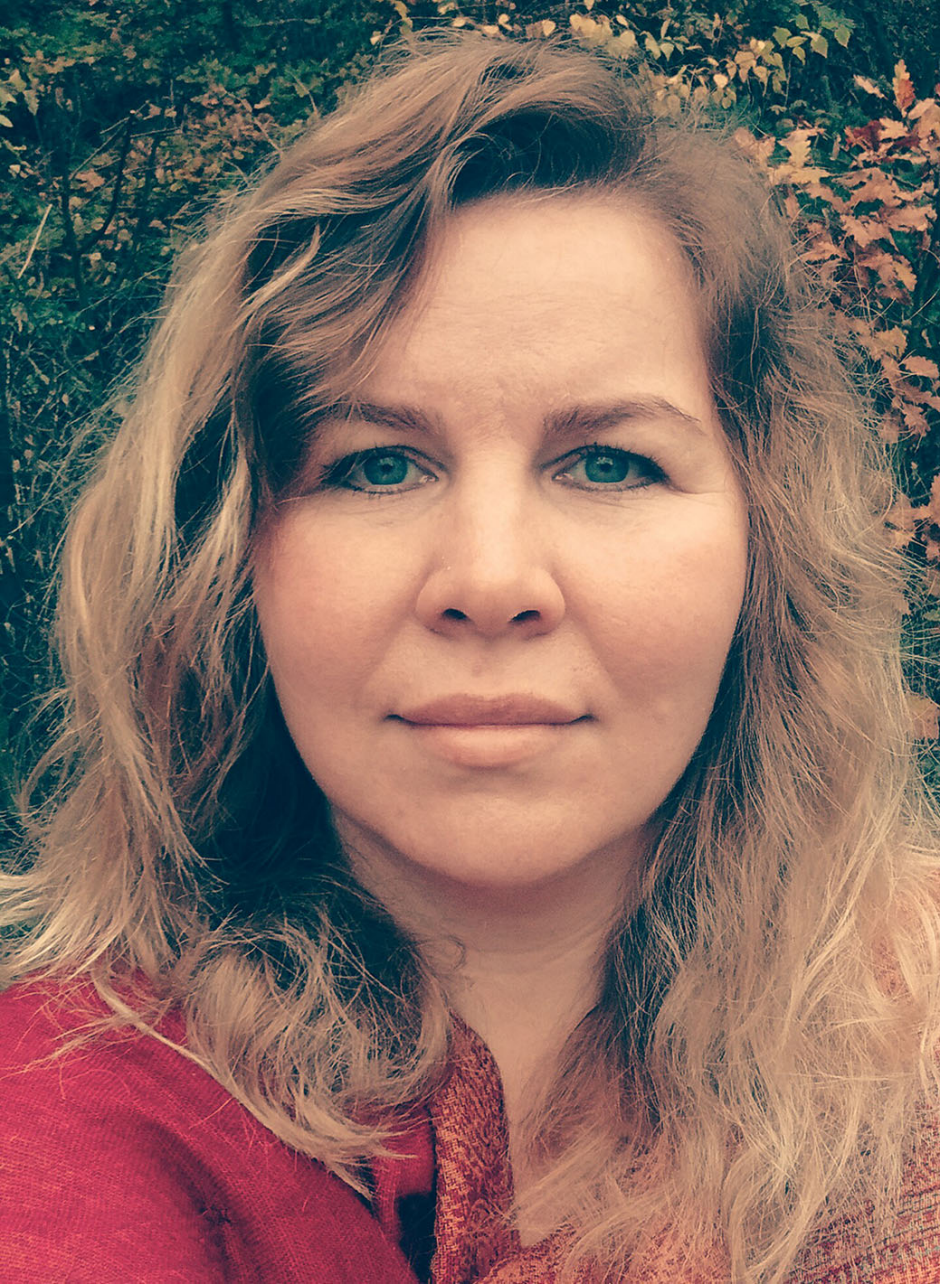


**Monika Baxa**

**How would you explain the main findings of your paper to non-scientific family and friends?**

Huntingtons's disease (HD) is a devastating neurodegenerative disorder characterized by motor disturbances, cognitive decline and personal changes. Although the genetic cause of the disease has been known for more than 25 years, there remains no cure for HD to date. Each of the prospective therapeutic treatments needs to be investigated for safety, tolerability and efficacy. Mouse and rat models are helpful for examination of the pathological mechanisms of HD, but they are not sufficient for completion of preclinical testing. Therefore, we generated a transgenic HD minipig to overcome the gap between rodents and humans. However, the generation of a transgenic animal does not mean that a disease model is generated. The phenotype observed in transgenic animals needs to correlate with the disease manifestation in patients. The more the model simulates the disease symptoms the better it is for translational research, as the safety and efficacy of the cure can be more finely evaluated. In this study, we demonstrated an HD-like clinical phenotype in our TgHD minipig model.

**What are the potential implications of these results for your field of research?**

Our TgHD minipig model may push forward preclinical trials, providing better preclinical outcome, including safety, tolerability, biodistribution, longitudinal investigation and efficacy of novel therapeutic approaches, and thus it could help to increase translatability to human medicine. Gene-lowering therapy using a microRNA approach showed encouraging results in our minipigs in a short 3-month survey (Evers et al., 2018). It was followed by approval for testing this strategy in phase-I and -II clinical trials by the Food and Drug Administration and the European Medicines Evaluation Agency. The most prominent clinical syndrome of HD is motor impairment. However, brain degeneration starts several years earlier. Scientists consider the late state of the preclinical stage of the disease a desirable period in which to start the application of therapy. Slow disease progression in our model could be suitable for investigation of the preclinical symptoms of HD.

“The main advantages of this model are its large brain size, long lifespan and anatomical similarities to humans, which enable the application of procedures and equipment commonly used in human medicine.”

**What are the main advantages and drawbacks of the model system you have used as it relates to the disease you are investigating?**

The main advantages of this model are its large brain size, long lifespan and anatomical similarities to humans, which enable the application of procedures and equipment commonly used in human medicine. Moreover, drug delivery to the large brain and its effect(s) can be monitored; for example, using magnetic resonance imaging, magnetic resonance spectroscopy or positron emission tomography. As a disadvantage, I consider the fact that, contrary to changes occurring in the brain, the changes in behavioral, motor and cognitive functions are not so unequivocally corresponding to those of humans. For example, minipigs are tetrapods, which helps them to maintain balance and makes them more stable in movement in comparison to humans.

**Describe what you think is the most significant challenge impacting your research at this time and how will this be addressed over the next 10 years?**

HD is caused by prolonged repetition of glutamines in the huntingtin gene. That means that, except for the repetitive sequence of CAG triplets, there is no difference between mutant and native huntingtin gene. Huntingtin protein is involved in many cellular pathways and it could be risky to silence it at all. Therefore, it is challenging to lower/silence the expression of mutant huntingtin explicitly. A potential solution may be single-nucleotide polymorphisms, which could enable specific targeting of mutant huntingtin. The next challenge is distribution of a potential drug to the brain because the brain-blood barrier limits penetration of larger molecules. There are some studies investigating intrathecal administration ­(administration of the drug to the brain via the cerebrospinal fluid). Next, it would be great if there could be found a way for oral administration of the drugs.

“The feeling of belonging to a collaborative scientific community may be a great motivation for all of us.”

**What changes do you think could improve the professional lives of early-career scientists?**

Networking. For early-career scientists, it is very important to meet professionals in their research topics in order to have a chance to learn from experienced scientists, to build collaborations, to present results and to look for real application of the results in life. Early-career scientists should be given opportunities to be educated in international internships and summer/winter school/training, as well as to attend vocational courses and conferences. The feeling of belonging to a collaborative scientific community may be a great motivation for all of us.

**Figure:**
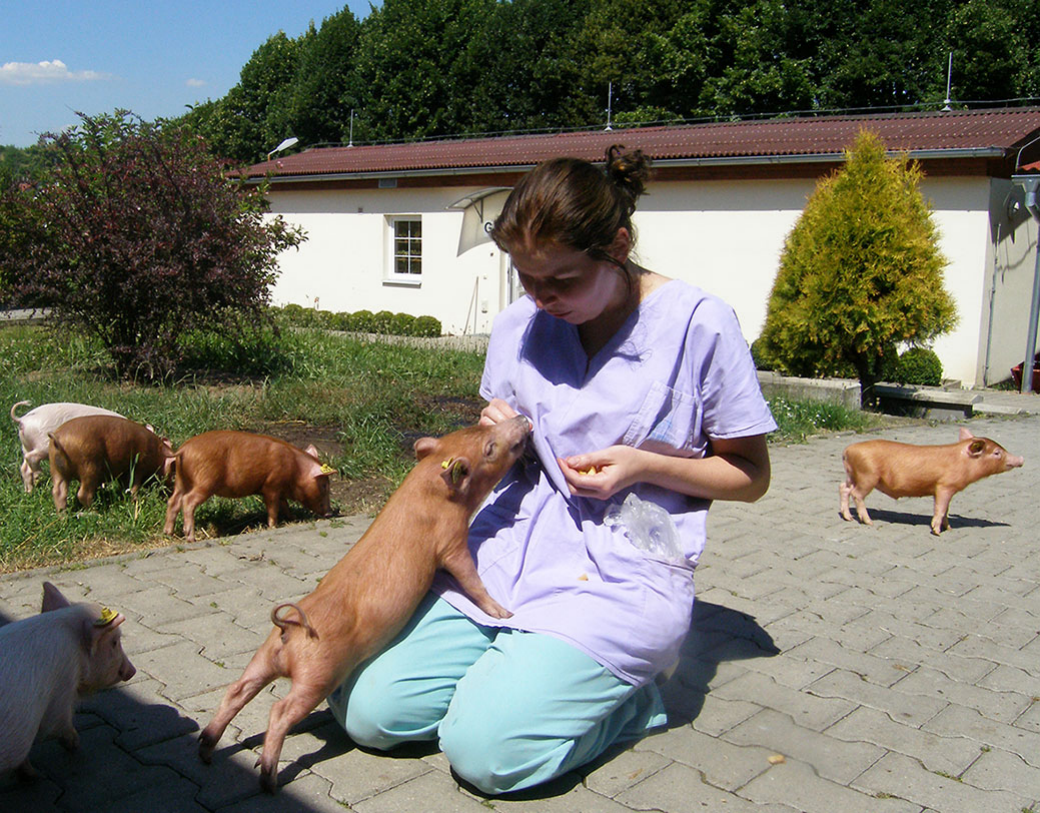
**Three-month-old TgHD minipigs during handling training.**

**What's next for you?**

I am finishing my PhD studies and planning to continue as a postdoctoral fellow in the HD field. I wish to energize the Slovak Huntington Association, which may help to improve the quality of life for HD patients in the Slovak Republic.
